# Effects of a Raisin Supplement on Cognitive Performance, Quality of Life, and Functional Activities in Healthy Older Adults—Randomized Clinical Trial

**DOI:** 10.3390/nu15122811

**Published:** 2023-06-20

**Authors:** María José Rodrigo-Gonzalo, Susana González-Manzano, María Carmen Pablos-Hernández, Roberto Méndez-Sánchez, Begoña Ayuda Duran, Jesús González-Sánchez, Fausto Barbero-Iglesias, Ana María González-Paramás, José Ignacio Recio-Rodríguez

**Affiliations:** 1 Grupo de Investigación de Polifenoles (GIP-USAL), Universidad de Salamanca, E-37007 Salamanca, Spain; mjoserodrigo@usal.es (M.J.R.-G.); bego_ayuda@usal.es (B.A.D.); paramas@usal.es (A.M.G.-P.); 2 Facultad de Enfermería y Fisioterapia, Universidad de Salamanca, E-37007 Salamanca, Spain; ro_mendez@usal.es (R.M.-S.); jesusgonzsan@usal.es (J.G.-S.); fausbar@usal.es (F.B.-I.); donrecio@usal.es (J.I.R.-R.); 3 Grupo de Fisioterapia, Recuperación Funcional y Ejercicio Terapéutico del Instituto de Investigación Biomédica de Salamanca (IBSAL), E-37007 Salamanca, Spain; 4 Hospital Universitario de Salamanca, Universidad de Salamanca, E-37007 Salamanca, Spain; carmenpablos@usal.es; 5 Unidad de Investigación en Atención Primaria de Salamanca (APISAL), Instituto de Investigación Biomédica de Salamanca (IBSAL), Red de Investigación en Cronicidad, Atención Primaria y Promoción de la Salud (RICAPPS), E-37007 Salamanca, Spain

**Keywords:** flavonoids, flavanols, raisins, phenolic compounds, cognitive function, cognitive performance, quality of life and functional activities

## Abstract

The objective of this study was to evaluate the effects of consuming 50 g of raisins on cognitive performance, quality of life, and functional activities in healthy older adults. This is a parallel randomized controlled clinical trial, in which 80 subjects over 70 years of age participated. For 6 months, the intervention group (IG; *n* = 40) consumed 50 g of raisins per day added to their usual diet, whereas the control group (CG; *n* = 40) received no supplement. All variables were measured at baseline and at 6 months. Cognitive performance assessed with the Montreal Cognitive Assessment (MOCA) test shows a difference of 3.27 points (95% CI 1.59 to 4.96), *p* ≤ 0.001, favorable to the IG, after the intervention. Among the cognitive performances, an improvement is observed in the IG in orientation, assessed both with the MOCA test 0.49 (95% CI 0.10 to 0.87), *p* = 0.014, and with the Mini-Mental State Examination (MMSE) test, 0.36 (95% CI 0.02 to 0.70), *p* = 0.038. In visuospatial/executive capacity and in language, improvements were also observed in the IG, 1.36 (95% CI 0.77 to 1.95), *p* = 0.001, and 0.54 points (95% CI 0.12 to 0.96), *p* = 0.014, respectively. Immediate and delayed recall, assessed with the Rey Auditory Verbal Learning Test, improved in the IG. In addition, the IG showed a better quality of life and greater autonomy in instrumental activities of daily living after 6 months. No significant changes were observed in the rest of the variables analyzed. Therefore, the consumption of 50 g of raisins produces a slight improvement in cognitive performance, quality of life, and functional activities in the elderly.

## 1. Introduction

Diet is a determining factor for health, populations that consume a diet rich in fruits and vegetables have a lower incidence of inflammatory, neurodegenerative, and cardiovascular diseases compared to populations with lower intakes of these foods. A Mediterranean-style diet, with a high content of phytochemicals, including polyphenols, is associated with a lower degree of inflammation biomarkers and a protective role in cerebrovascular and cardiovascular events [1].

Some phenolic compounds may influence the pathological mechanisms underlying cognitive degeneration. These compounds could act not only as antioxidant and anti-inflammatory agents, but also as modulators of molecular mechanisms that play a role in the development of chronic degenerative diseases. Epidemiological studies suggest that nutritional intervention may prevent age-related cognitive decline, especially when the diet contains more than one bioactive nutrient [2]. The regular intake of foods rich in polyphenols, and especially flavonoids, could help prevent or mitigate the progression of different neurological diseases [3].

Among the foods rich in polyphenols is the grape, whose moderate consumption is believed to have benefits at a cognitive level. Neuroprotective benefits have been attributed to moderate consumption of grape polyphenols [4]. One serving of unpeeled table grapes (200 g; flame seedless) could provide up to 72 mg of total phenols, including anthocyanins (cyanidin, malvidin, peonidin, petunidin, and delphinidin), flavanols (catechin, epicatechin, procyanidins B1, B2, and C1, and gallocatechin), and, to a lesser extent, hydroxycinnamic acid (caffeic acid and *p*-coumaric acid), stilbene (resveratrol), and flavonols (especially rutinoside and quercetin glucoside) [5]. This diversity of compounds makes grapes, especially red grapes, excellent candidates for testing the role of dietary polyphenols in health. The phenolic profile of raisins is different from that of grapes, due to the hydrolysis and oxidation processes that occur in them. In recent years, research has intensified, demonstrating that raisins have a low to moderate glycemic index, a low insulin index [6], and provide a feeling of satiety. In addition, its consumption has also been linked to a significant reduction in blood pressure, cholesterol, low-density lipoproteins, triglycerides, serum oxidized fatty acids, and proinflammatory cytokines [7]. Therefore, raisins could significantly reduce the risk of developing diabetes or cardiovascular disease.

The effect on cognitive functions of interventions that use supplements or foods rich in polyphenols in healthy older adults indicated insufficient evidence regarding this supplementation’s neuroprotective and anti-inflammatory effects. However, the findings from individual studies included in this systematic review suggest that polyphenol-rich supplementation may improve some isolated cognitive functions. Furthermore, the beneficial effect of polyphenols seems to depend on the ingested dose and bioavailability. The results suggest that at least an intermediate dose of polyphenols (≥500 mg) and intermediate (~9%) to high (43%) bioavailability rates are required to cross the blood–brain barrier and exert a significant effect on cognitive health. At a cognitive level, no studies directly related to raisins have been found, but there is literature that demonstrates the cognitive benefits of flavonols, flavanols, and stilbenes, families of polyphenols present in raisins. Specifically, in supplementation with flavanols or stilbenes, an improvement in attention, psychomotor speed, delayed memory, and word recognition is observed. The synergistic action of flavanols and flavonols improves visual memory in patients without cognitive pathology and working memory in patients with pathology. The results are influenced by the type of polyphenol, the duration, and the evaluation test used [8].

The objective of this study is to analyze the effects of adding 50 g of Málaga muscatel raisins to the usual diet on cognitive performance, quality of life, and functional activities in healthy older adults.

## 2. Materials and Methods

### 2.1. Design

This is a controlled and randomized clinical trial with two parallel groups. Participants were recruited in two urban health centers in Salamanca and Zamora, Spain, through consecutive sampling. The study was carried out between September 2021 and April 2023 at the Faculty of Nursing and Physiotherapy of the University of Salamanca.

The clinical trial has been registered on clinicaltrials.gov provided by the US National Library of Medicine as NCT04966455, registration date 1 July 2021. The results reported in this manuscript are primary study results. The trial protocol has been published [9].

### 2.2. Study Participants and Recruitment

**Figure 1:**
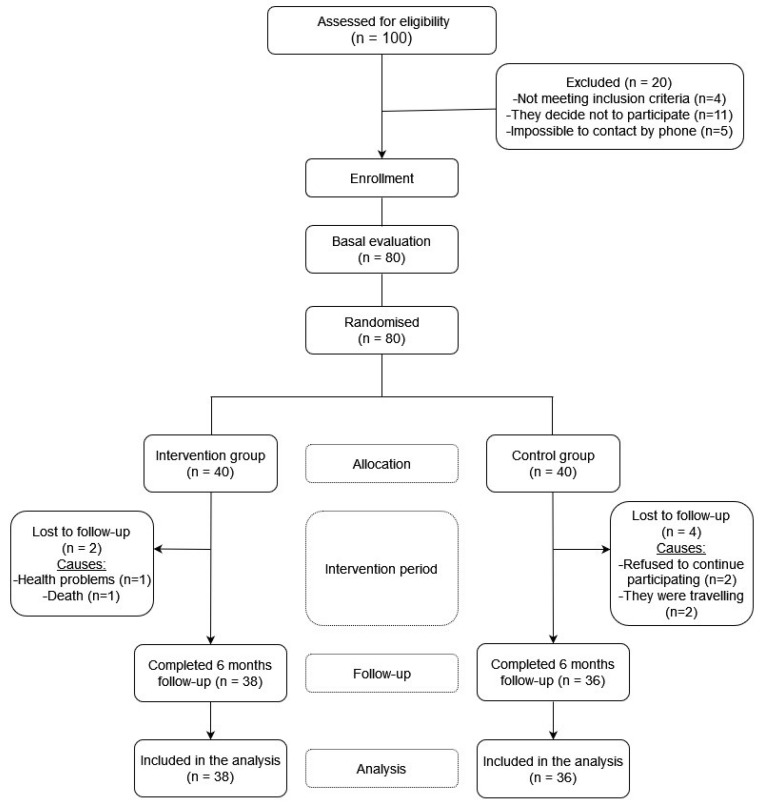
Study flowchart.

From the primary care services of the health centers, 100 volunteers were selected, but finally, 80 participated in the study (10 people decide not to participate when the main researcher contacted them by phone to make an appointment for the initial interview, 1 person attended the initial interview but finally decided not to participate, 1 person obtained less than 24 points in the Test Mini-Mental State Examination (MMSE), 3 people were interested in the study but were not able to travel to the faculty and 5 people were not possible to contact by phone to make an appointment for the interview) (Figure 1).

The inclusion criteria were: people >70 years of age (men and women), score on the MMSE Test ≥ 24 points, being able to attend the place where the evaluations were carried out, and signing the informed consent. The exclusion criteria were: coronary or cerebrovascular disease, advanced renal or hepatic disease, severe mental illness, diabetes mellitus, grade II or higher heart failure, moderate or severe chronic obstructive pulmonary disease, oncological disease diagnosed in the last 5 years under treatment or terminal situation, morbid obesity (BMI ≥ 40 kg/m^2^), intolerance and/or allergy to any of the components of the raisins or any other circumstance at the discretion of the researchers.

### 2.3. Sample Size

The sample size was calculated to detect a difference of ≥2 words as statistically significant in the categorical fluency test. For this objective, the inclusion of 80 participants is required, 40 for each group. The estimation has taken into account the standard deviation of 4.75 and the correlation coefficient between the initial and final measurement shown by the results of the work by Garcia-Yu et al. [10], considering an alpha and beta risk of 0.05 and 0.2, respectively, and an estimate of withdrawals of 10%.

### 2.4. Randomization and Study Procedures

Participants had an initial visit and a follow-up visit 6 months after the initial assessment (Figure 1). The intervention group underwent 5 additional follow-up visits after the initial visit (months 1, 2, 3, 4, and 5). During these visits, the necessary raisins were delivered until the next visit and the raisin intake recording calendar for the following month. In addition, in these replacement visits, the participants gave the research staff the calendar with the record of the intake of raisins carried out the previous month.

After the baseline evaluation, the participants were randomly assigned to the intervention group (IG) (*n* = 40) or the control group (CG) (*n* = 40). The sequence was generated by an independent researcher using the Epidat 4.2 program (Department of Health, Government of Galicia, Spain) with a ratio of 1/1. Participants received their allocation number based on the order of the initial visit. To ensure that blinding was maintained as well as possible, participants were instructed not to reveal which treatment they had been randomized to while being interviewed by the blinded assessor. Due to the characteristics of the intervention, it was not possible to blind all participants so the research was a single-blind study. To minimize cross-contamination between groups, the investigator performing the evaluations was different from the one performing the replenishment of grapes for the IG.

### 2.5. Intervention

All study participants (CG and IG) prior to randomization and after the baseline visit received brief nutritional counseling with recommendations on a balanced diet (see Supplementary Material).

The IG participants received Málaga muscatel raisins (50 g/day) to add this supplement to their usual diet. In addition, they were given a calendar to record their consumption. No specific instructions were given regarding the time of intake, nor if its consumption had to be with or without other foods, they were decisions made by each participant according to their preference.

The daily nutritional contribution of 50 g of Málaga muscatel raisins, as indicated on the label, is 150 kcal, 36 g of carbohydrates of which 14 g are sugar, 1 g of protein, 0.25 g of fat, and 0.02 g of salt.

The analysis of the polyphenolic composition of the Moscatel de Málaga raisin grape was carried out with a mass spectrometer (HPLC-DAD-ESI/MS) using a high-performance liquid chromatography technique and quantified by comparing the chromatographic peaks against prepared calibration curves with the external standards of each compound. Table 1 shows the polyphenolic profile analyzed, which is summarized in a contribution of 9.48 mg of polyphenols per 50 g of raisins, corresponding 1.00 mg of hydroxycinnamic acids, 6.76 mg of flavanols and 1.72 mg of flavonols.

### 2.6. Adherence to the Intervention

By recording the daily intake of raisins that each IG participant took, the percentage of compliance with respect to the theoretical maximum was calculated, which is what adherence represents.

### 2.7. Variables and Measuring Instruments

The main variable of the study is cognitive performance measured by various tests (Montreal Cognitive Assessment (MOCA), Mini-Mental State Examination test (MMSE), Rey Auditory Verbal Learning Test, and verbal fluency). Functional activities were also measured through the Pfeffer Functional Activities Questionnaire (FAQ) and quality of life through the EuroQol Questionnaire and the World Health Organization Quality of Life Instrument (WHOQOL) in its adaptation of WHOQOL-AGE.

Montreal Cognitive Assessment (MOCA) [11]: The MOCA test that has been used is the version validated in Spanish and analyzes different dimensions of cognitive performance such as executive functions, short-term and working memory, attention and concentration, visuospatial capacity, orientation, and language. The highest score that can be achieved is 30 points. A score ≥ 26 points suggests normal cognitive performance. In calculating the score, an additional point is added to people over 12 years of age who are in school.

Mini-Mental State Examination (MMSE) [12]: The MMSE evaluates different dimensions of cognition such as memory, comprehension, naming, attention, repetition, reading, or orientation. Each individual obtains a score between 0 and 30 points considering the value of 24 points as the cut-off point for normal cognitive performance.

Rey Auditory Verbal Learning Test [13]: In this test, the individual is asked to remember a series of words that the evaluator reads several times. With this, immediate and delayed verbal memory is analyzed.

Verbal fluency [14]: The participant is asked to list as many animal names as possible for one minute.

Pfeffer Functional Activities Questionnaire (FAQ): The test designed by Pfeffer [15,16] assesses an individual’s ability to perform instrumental activities of daily living such as preparing food, keeping abreast of community events, visiting friends and safely going out of the neighborhood alone, managing money, understand and read news or remember important dates, among others. Each of the 11 items that make up the questionnaire is scored between 0 (totally independent for that activity) and 3 (full dependence).

World Health Organization Quality of Life Instrument (WHOQOL) in its adaptation to the elderly population WHOQOL-AGE [17]: In this study, we have used the short version of this questionnaire that has been validated in a population over 50 years of age. The total score of this questionnaire is between 0 and 100 points, with a higher score corresponding to a better quality of life.

EuroQol Questionnaire [18,19]:

The EuroQol questionnaire assesses quality of life using two instruments: the EuroQol-5D (EQ-5D) and the EuroQol Visual Analogue Scale (EQ-VAS). The EuroQol-5D (EQ-5D) analyzes five dimensions related to the presence of pain, anxiety and depression, self-care, mobility problems, and problems in carrying out activities of daily living [20]. For its part, the EuroQol Visual Analogue Scale (EQ-VAS) analyzes the general state of health perceived by each person, ranging from 0 to 100, the latter corresponding to the best possible state of health.

Data collection procedure, data management, and monitoring.

Data from the baseline assessment and the 6-month visit were collected by a specially trained nurse. Each study participant was identified with a unique code, which corresponded to the data collected at each of the visits. All measurements were compiled in a data collection notebook and kept in a safe place at the faculty. A database was created with all the values collected, which could only be accessed by the study researchers.

### 2.8. Ethical Considerations

The study was approved by the Salamanca Health Area Clinical Research Ethics Committee (CEIm Code: PI 2020 10 578). The participants, before the start of the study, were informed of the objectives of the project and the risks and benefits of the explorations. Subsequently, they signed the informed consent in accordance with the Declaration of Helsinki [21].

All the data collected in the study have been processed following the regulations in force in Spanish territory regarding the protection of data in biomedical research: Organic Law 3/2018 on the protection of personal data, Law 14/2007 on biomedical research, and European Regulation 2016/679 of data protection.

### 2.9. Statistical Analysis

Statistical analyses were performed as described in the study protocol [9], using the statistical program SPSS version 26.0 (IBM Corporation, Armonk, NY, USA).

The normality of the variables collected in the study was analyzed using the Kolmogorov–Smirnov test and in cases that did not follow statistical normality, the corresponding non-parametric tests were used. The descriptive analysis presents the results of the categorical variables with the *n* and the percentage (%), whereas the continuous variables are expressed with the mean and the standard deviation. The chi-square test was used to analyze the association between two categorical variables from two or more categories. Using the Student’s *t*-test (Mann–Whitney U) for independent samples, the means between two groups were compared, evaluating the change within the same group with the Student’s *t*-test for paired data (Wilcoxon test).

The results of the primary variables (cognitive performance) and secondary variables (quality of life and functional activities) were analyzed by intention to treat (including all subjects who were randomized according to the assigned group). The effect of the intervention on the variables evaluated was analyzed with an analysis of covariance-ANCOVA, with an adjustment for those variables that showed significant differences between both groups in the initial evaluation (withdrawal time). The “center” variable was also included in the analysis model because the study was conducted in two different health centers.

## 3. Results

### 3.1. Characteristics of the Study Population

The study population consisted of 80 participants (IG = 40, CG = 40), 66.3% were women, with an average age of 76.7 (SD 4.6) years. A total of 61.3% of the participants were married, with the number of cohabitants in the house being 1.9 ± 0.7, including the participant. A total of 46.3% had finished their primary studies, whereas 23.8% had completed university studies. Regarding the level of physical activity, 33.8% maintained vigorous activity, 58.8% moderate, and 7.5% were sedentary. There was a difference in terms of the time that had elapsed since their retirement, IG 11.3 ± 5.4 years and CG 14.5 ± 6.4 years, *p* = 0.037 (Table 2).

Mean adherence to the intervention was 89.4% ± 21.1, with the maximum being 100% (180 days).

### 3.2. Changes in Cognitive Performance

Cognitive performance was measured using the MOCA test, the MMSE, Rey Auditory Verbal Learning Test, and verbal fluency. In all the tests, except for verbal fluency, we found some significant improvement after the intervention.

The MOCA test shows a significant improvement in the IG. The variation in the IG between the baseline to the post-dose assessments is 2.81 (95% CI 1.72 to 3.90), in the CG −0.46 (−95% CI 1.70 to 0.77), which is a difference of 3.27 (95% CI 1.59 to 4.96) points (*p* ≤ 0.001). Making a detailed analysis of each one of the cognitive abilities that the test evaluates, we observe that there are also significant differences between the IG and the CG in the visuospatial-executive capacity, 1.36 (95% CI 0.77 to 1.95) points (*p* ≤ 0.001), language, 0.54 (95% CI 0.12 to 0.96) points (*p* = 0.014), and orientation, 0.49 (95% CI 0.10 to 0.87) points (*p* = 0.014) (Table 3).

In the overall score of the MMSE test, we did not observe significant differences between the two groups. However, if we look at each of the items, we find significant differences in time orientation. The change between the baseline to the post-dose assessments in the IG was 0.01 (95% CI −0.21 to 0.23), whereas in the CG it was −0.35 (95% CI 0.60 to 0.10), which implies a difference of 0.36 (95% CI 0.02 to 0.70) points (*p* = 0.038) between the two groups. The results of spatial orientation, memory and comprehension, reading, writing, and drawing of the IG improved numerically at the 6-month interview, but the difference between the CG and the IG is not significant. The item of memory and fixation does not vary in either of the two evaluations, neither in the IG nor in the CG. We observed that in the CG spatial orientation, memory and comprehension, reading, writing, and drawing have worsened in the 6-month evaluation compared to the baseline visit, but the difference between the CG and the IG is not significant either (Table 4).

In the Rey Auditory Verbal Learning Test (RAVLT) we observed significant differences between both groups (IG and CG) in both immediate (RAVLT-IR) and delayed (RAVLT-DR) recall. Regarding immediate recall, in the IG there is a change of 1.65 (95% CI 1.20 to 2.10) words from the baseline visit to the final visit, whereas in the CG it is 0.45 (95% CI −0.06 to 0.95). Therefore, the difference between both groups is 1.2 (0.51 to 1.89) words (*p* = 0.01). In the delayed memory, the change we observed in the IG is 2.08 (95% CI 1.28 to 2.89) and in the CG it is 0.38 (95% CI −0.53 to 1.29), that is, it shows a difference of 1.7 (95% CI 0.46 to 2.94) words (*p* = 0.08) (Table 5).

### 3.3. Changes in the Quality of Life and Functional Activities

We measured quality of life with two instruments, the EuroQol 5-D Questionnaire and the World Health Organization Quality of Life Instrument (WHOQOL) in its WHOQOL-AGE version.

Through the EuroQol 5-D Questionnaire (Table 6), we observed that both in the IG and the CG, at the 6-month visit, the patients who reported not having problems walking increased with respect to the baseline visit. In the same way, more patients reported having no problems performing daily activities at the 6-month visit than at the baseline visit. Patients who reported being moderately anxious or depressed decreased in both groups. No differences were observed between groups in the EQ-5D-3L score or in the EQ-VAS, Table 7.

In the WHOQOL-AGE Quality of Life Scale (Table 7) we observed significant changes. In the IG the change that occurs between the initial and final interview is 1.21 (95% CI −0.13 to 2.55) points and in the CG it is −1.39 (95% CI −2.93 to 0.15). The difference between both groups is 2.61 (95% CI 0.52 to 4.70) points (*p* = 0.015).

With the Pfeffer functional activities questionnaire, we observed that there are significant positive changes in the IG. In this group the change that occurs throughout the study is −0.24 (95% CI −0.57 to 0.09) and in CG 0.69 (95% CI 0.32 to 1.05), with a difference between the two of −0.93 points (95% CI −1.44 to 0.43) (*p* ≤ 0.001). IG patients are more independent at the end of the study (Table 7).

## 4. Discussion

### 4.1. Discussion of Cognitive Performance Results

The results of this study show an improvement in “orientation”, assessed both in the MOCA test and in the MMSE test (temporal orientation), after daily consumption of 50 g of Málaga muscatel raisins. In addition, in the “visuospatial/executive” and “language” categories, also assessed with the MOCA test, a higher score was observed in the IG after 6 months. In the final score of the MOCA test, we also observed a significant improvement in the IG. With the Rey Verbal Auditory Learning test, we observed an improvement in the “immediate recall” and in the “retention memory” of a list of 15 words, in the IG subjects after a non-amnestic interference task. “Immediate recall” is understood as the number of words recalled on the first attempt after the researcher has read 15 words. The “retention memory” is the number of words correctly remembered 30 min after a non-amnestic interference task (maximum 15 words).

However, no relevant differences were found in the other items evaluated with the Mini-Mental State Examination test (“spatial orientation”, “memory and fixation”, “attention and calculation”, “memory”, “naming and repetition” and “comprehension”, reading, writing and drawing”) nor with the MOCA test (“identification”, “memory”, “attention”, “concentration”, “similarities” and “delayed recall”). No differences were observed in verbal fluency either, used in the estimation of the sample size.

The effect of raisins on cognitive performance has not been previously studied. However, an abundance of literature shows that polyphenolic compounds, mainly the families of flavonols, flavanols, and stilbenes, could have a beneficial effect at a cognitive level. These families of compounds are well represented in raisins, as shown in Table 1. The exact mechanism by which polyphenols provide such positive effects is not exactly known, but the consumption of compounds from the families of flavanols and stilbenes, present in a large number of foods of vegetable origin, such as tea, cocoa, grapes, and red wine, has been shown to improve the bioavailability of nitric oxide [22,23,24]. This compound produces an increase in vasodilation and with it improves cerebral perfusion. Therefore, it is believed that they could counteract neuroinflammation. Underlying mechanisms involving the microbiota-gut-brain axis have also been described [25,26,27].

Following a review on this topic, attention was found to improve with a flavanol 900 mg/day supplement for 2 months [28], with a flavanol 993 mg/day drink for 2 months [29], and with 1000 mg /day of resveratrol for 3 months [30] in healthy patients. In these studies, the Trail Making Test (TMT A and B) and the Wechsler Adult Intelligence Scale III (WAIS III) were used as tools to measure cognition. An improvement in visual memory was also observed with a daily supplement of 900 mg cocoa and 138 mg epicatechin for 3 months in healthy subjects [31]. Attention and memory are items that are also evaluated with the MMSE test. However, in the total score of said test, the investigations mentioned above [28,29], like our study, do not show post-intervention improvement. On the other hand, the TMT A and B test is similar to one of the tests used in the “visuospatial/executive” item of the MOCA test, and in which we did observe a significant improvement in the IG after the intervention in our study.

For all these reasons, we can say that the accuracy of the tool used to assess cognition in this type of study is key to making an adequate evaluation. There are several studies in which the MMSE test is used, and like us, they do not observe changes in the final score after the intervention, and yet they do with other tests [28,29,32,33]. As Hernando et al. already pointed out, the MMSE test is a tool with limitations to be used in research [34].

Similar results to those observed in our work have been described by Lopresti et al. in older adults with mild cognitive impairment. The IG obtained significant improvements in “visuospatial learning”, as well as in other cognitive functions such as “speed of information processing” after a daily supplement of 150 mg of grape and blueberry polyphenol extract twice daily for 6 months [35].

Witte et al. demonstrated higher scores in delayed recall and word recognition after daily supplementation with 200 mg of resveratrol and 320 mg of quercetin for 6 months [36]. In our study, we also observed an improvement in delayed recall, as well as in immediate recall, but we did not observe it in word recognition. This difference may be influenced by the number of polyphenols supplemented daily (lower in our study) and by the average age of the patients (older in our study).

With the Rey Auditory Verbal Learning Test, both our study and Witte et al. observed improvement after the intervention in healthy patients. However, when analyzing the influence of a daily supplement of 200 mg of resveratrol and 350 mg of quercetin for 26 weeks (very similar to the product used by Witte et al., who used 200 mg of resveratrol and 320 mg of quercetin for 6 months) on patients with cognitive pathology, no changes are observed [37]. Therefore, this could lead us to think that a similar intervention does not affect patients with cognitive pathology in the same way as it does in healthy patients.

In general, foods of plant origin contain different polyphenolic compounds with different concentrations. In addition, the total content of polyphenols in foods is influenced by edaphoclimatic factors and culinary preparation. Other foods rich in polyphenols that have been shown to improve cognitive aspects are, among others, cocoa [38,39], olive oil [40,41], nuts [40], fish [42,43,44], or tea [33].

This study, like other articles mentioned, can also affirm that flavonoids, present in raisins, may be related to better cognitive performance.

### 4.2. Discussion of the Results of the Quality of Life and Functional Activities

The progressive aging of the population implies an increase in chronic diseases and dependency, which, in turn, reduces the quality of life. For this reason, it is important to look for interventions, especially non-pharmacological ones, that can improve our quality of life [45].

The results of this clinical trial show that daily consumption of 50 g of Málaga muscatel raisins produces an improvement in the WHOQOL-AGE Quality of Life Scale score. The IG improves by 2.61 points with respect to the CG.

Currently, we find little previous literature that directly evaluates the quality of life related to the intake of polyphenols. To the best of our knowledge, there are no similar studies with raisins with which to compare the results, regarding quality of life and functional activities.

A clinical trial carried out on elderly people showed that daily consumption of a natural drink made from cocoa powder (rich in flavonoids) improved the quality of life score measured through the EuroQol 5-D scale. Specifically, an improvement was observed in mobility problems and pain/discomfort [46]. However, another clinical trial states that 10 g of chocolate rich in cocoa (99%) per day in postmenopausal women, for 6 months, could have a slight impact on the perception of their health, although without modifying the health-related quality of life [47]. There are also some studies that mention a better quality of life in patients when ingesting polyphenols, but without specifically measuring it, but rather as a secondary consequence. Cases and collaborators demonstrated that 900 mg of a treatment rich in polyphenols extracted from fruits and vegetables characteristic of the Mediterranean diet, in obese patients, improves anthropometric and blood parameters, thus improving the metabolic aging process and increasing their quality of life [48]. Romain et al. have shown an extract of polyphenols and yerba mate to improve the overall quality of life score [49]. However, we also found an article that found no significant changes in quality of life after supplementation with a fruit and vegetable concentrate [50]. In these last two articles, they used a test different from our study, they evaluated the quality of life through the SF-36 health questionnaire, used for the general population [51].

Gopinath et al. concluded that a higher quality diet is associated with a higher quality of life and better functional capacity [52]. The Mediterranean diet is rich in omega 3 fatty acids, antioxidants, and polyphenols. It is considered a cardioprotective and neuroprotective diet. In addition, it has been shown that adherence to the Mediterranean diet for 4 years is associated with a higher quality of life score assessed by the SF-36 health questionnaire [53]. However, focusing on grapes, O’Connor et al. demonstrated that daily consumption of grapes for 6 weeks (2 servings of ¾ of a grape serving) did not significantly influence fitness or work capacity. We should note that this study was conducted with young adults, unlike our study [54].

### 4.3. Strengths and Limitations

This trial has a larger number of participants compared to other studies looking at the effect of polyphenols on cognitive performance [30,31,32,33,36,37,55,56]. In addition, the study intervention consisted of supplementing the usual diet with a quantity of commercial raisins, with unchangeable specific characteristics. This provides a real clinical context, being able to evaluate both the potential benefits and harms of the intake of this product. Therefore, this makes the results of this trial more accessible than other studies that use off-the-shelf compounds, specifically crafted for research, and are not available in a real-world setting. [29,30,32,36,57].

Regarding the limitations of this study, it should be noted that it was not possible to blind the participants due to the nature of the intervention, although blinding of the investigators was ensured in the measurements and in the statistical analyses. Furthermore, the tool used to assess the nutritional composition of the habitual diet, the 3-day dietary record, does not provide exact data on dietary polyphenols. Despite this, we can assume that randomization would have balanced the groups with respect to dietary intake. However, it should be taken into account in future trials. Another limitation of our study is that the amount of polyphenols administered in the IG contains a low concentration of polyphenols, compared to other studies. On the other hand, we must bear in mind that repeating the same tests 6 months after the baseline visit may have a learning component for both groups, despite the fact that this is the recommended period of time to avoid the learning effect.

## 5. Conclusions

The results of this study suggest that the supplement of 50 g of Málaga muscatel raisins slightly improves cognitive performance, specifically orientation, visuospatial/executive capacity, language, the overall MOCA test score, and immediate and delayed recall. In addition to showing a slight improvement in quality of life and functional activities. These are positive data, but more research is needed to know exactly the mechanism of action of polyphenols on cognitive performance.

## Figures and Tables

**Table 1:** Polyphenol composition of 50 g of Málaga moscatel raisins used in the intervention.

Compounds	Quantity, mg/50 g
Coumaric acid	0.42
Ferulic acid	0.22
Caffeic acid	0.36
Catechin	0.22
Epicatechin	0.81
Procyanidin dimer	1.92
Procyanidins trimers	2.72
Galloyl dimer procyanidins	0.86
Epigallocatechin gallate	0.23
Quercetin	0.28
Quercetin glucoside	0.98
Quercetin methyl glucoside	0.37
Kaempferol glucoside	0.37
Total polyphenols	9.48

**Table 2:** Baseline characteristics of the study population.

	Intervention Group (*n* = 40)	Control Group (*n* = 40)	*p* Value
Age (years)	76.3 (4.6)	77.0 (4.6)	0.511
Females (*n*, %)	30 (75.0%)	23 (57.5%)	0.098
Marital status (*n*, %)			0.775
Single	1 (2.5%)	2 (5.0%)	
Married	23 (57.5%)	26 (65.0%)	
Widower	13 (32.5%)	10 (25.0%)	
Separated or divorced	3 (7.5%)	2 (5.0%)	
Number of cohabitants	1.7 (0.5)	2.0 (0.9)	0.087
Educational level (*n*, %)			0.124
University studies	12 (30.0%)	7 (17.5%)	
Middle or high school	14 (35.0%)	10 (25.0%)	
Elementary school	14 (35.0%)	23 (57.5%)	
None	0 (0%)	0 (0%)	
Number of children	2.1 (1.1)	2.7 (2.1)	0.108
Retired time (years) *	11.3 (5.4)	14.5 (6.4)	0.037
Physical activity level			0.221
Sedentary	5 (12.5%)	1 (2.5%)	
Moderately active	23 (57.5%)	24 (60.0%)	
Vigorously active	12 (30.0%)	15 (37.5%)	
Adherence to the intervention, %	89.4 (21.1)		

Categorical variables are expressed as *n* (%) and continuous variables as mean ± standard deviation. Physical activity level according to the criteria established by the International Questionnaire of Physical Activity (IPAQ). * Statistically significant differences (*p* < 0.05).

**Table 3:** Changes in cognitive performance as assessed through the Montreal cognitive assessment-MOCA test.

	Intervention Group (*n* = 40)			Control Group (*n* = 40)				
	Baseline	6 Months	Change (6 Months-Baseline)	Baseline	6 Months	Change (6 Months-Baseline)	Difference (IG-CG)	*p* Value
MOCA total score/30	21.39 (3.09)	24.39 (3.44)	2.81 (1.72 to 3.90)	20.64 (3.84)	20.39 (4.42)	−0.46 (−1.70 to 0.77)	3.27 (1.59 to 4.96)	0.000
Visuospatial-executive/5	3.37 (1.10)	4.29 (0.73)	1.00 (0.61 to 1.38)	3.72 (1.06)	3.39 (1.27)	−0.37 (−0.80 to 0.07)	1.36 (0.77 to 1.95)	0.001
Naming/3	2.68 (0.53)	2.66 (0.48)	0.02 (−0.20 to 0.23)	2.44 (0.77)	2.67 (0.54)	0.16 (−0.08 to 0.41)	−0.15 (−0.48 to 0.19)	0.384
Attention/2	1.61 (0.64)	1.53 (0.65)	−0.08 (−0.35 to 0.19)	1.39 (0.60)	1.19 (0.71)	−0.27 (−0.57 to 0.04)	0.19 (−0.23 to 0.61)	0.371
Concentration/4	3.37 (0.94)	3.58 (0.64)	0.01 (−0.31 to 0.34)	3.22 (0.72)	3.33 (0.93)	0.21 (−0.16 to 0.57)	−0.19 (−0.69 to 0.31)	0.448
Language/3	1.74 (0.86)	2.16 (0.89)	0.45 (0.18 to 0.73)	1.28 (1.06)	1.25 (0.94)	−0.09 (−0.40 to 0.22)	0.54 (0.12 to 0.96)	0.014
Abstraction/2	1.74 (0.50)	1.84 (0.37)	0.12 (−0.09 to 0.33)	1.53 (0.74)	1.69 (0.62)	0.11 (−0.12 to 0.35)	0.01 (−0.32 to 0.33)	0.977
Delayed recall/5	1.11 (1.45)	2.50 (1.64)	1.22 (0.67 to 1.77)	1.39 (1.61)	1.64 (1.79)	0.39 (−0.23 to 1.01)	0.83 (−0.02 to 1.68)	0.056
Orientation/6	5.79 (0.41)	5.79 (0.41)	0.00 (−0.25 to 0.25)	5.64 (0.59)	5.31 (0.75)	−0.48 (−0.76 to −0.20)	0.49 (0.10 to 0.87)	0.014

MOCA: Montreal Cognitive Assessment. Values are means ± SDs and differences are means (95% CI). Intragroup comparison by the paired Student’s *t*-test. Intergroup comparison by the Student’s *t*-test. These values are adjusted for retired time and center. Results are based on ANCOVA.

**Table 4:** Changes in cognitive performance as assessed through the Mini-Mental State Examination-MMSE test.

	Intervention Group (*n* = 40)			Control group (*n* = 40)				
	Baseline	6 Months	Change (6 Months-Baseline)	Baseline	6 Months	Change (6 Months-Baseline)	Difference (IG-CG)	*p* Value
MMSE total score/30	28.55 (1.20)	28.76 (1.08)	0.23 (−0.28 to 0.74)	28.14 (1.55)	28.08 (1.40)	−0.25 (−0.82 to 0.32)	0.48 (−0.30 to 1.26)	0.222
Time orientation/5	4.74 (0.44)	4.79 (0.41)	0.01 (−0.21 to 0.23)	4.61 (0.65)	4.42 (0.60)	−0.35 (−0.60 to −0.10)	0.36 (0.02 to 0.70)	0.038
Spatial orientation/5	4.97 (0.16)	4.97 (0.16)	0.02 (−0.11 to 0.14)	4.86 (0.35)	4.83 (0.38)	−0.02 (−0.16 to 0.13)	0.03 (−0.17 to 0.23)	0.734
Registration/3	3.00	3.00	No change	3.00	3.00	No change	No change	
Attention and calculation/5	4.76 (0.49)	4.74 (0.76)	−0.01 (−0.22 to 0.19)	4.78 (0.49)	5.00 (0.00)	0.20 (−0.03 to 0.44)	−0.22 (−0.54 to 0.10)	0.182
Recall/3	2.50 (0.69)	2.66 (0.48)	0.17 (−0.13 to 0.47)	2.33 (0.76)	2.36 (0.87)	−0.03 (−0.37 to 0.31)	0.20 (−0.27 to 0.66)	0.394
Nomination and repetition/3	3.00 (0.00)	3.00 (0.00)	−0.00 (−0.05 to 0.04)	2.97 (0.17)	3.00 (0.00)	0.04 (−0.01 to 0.10)	−0.05 (−0.12 to 0.02)	0.188
Language/6	5.58 (0.55)	5.61 (0.50)	0.05 (−0.16 to 0.27)	5.58 (0.55)	5.44 (0.61)	−0.14 (−0.38 to 0.10)	0.20 (−0.13 to 0.53)	0.238

MMSE: Mini-Mental State Examination. Values are means ± SDs and differences are means (95% CI). Intragroup comparison by the paired Student’s *t*-test. Intergroup comparison by the Student’s *t*-test. These values are adjusted for retired time and center. Results are based on ANCOVA.

**Table 5:** Changes in cognitive performance assessed through verbal fluency, phonological fluency, and Rey Auditory Verbal Learning test.

	Intervention Group (*n* = 40)			Control Group (*n* = 40)				
	Baseline	6 Months	Change (6 Months-Baseline)	Baseline	6 Months	Change (6 Months-Baseline)	Difference (IG-CG)	*p* Value
Category fluency (words)	18.05 (4.01)	19.24 (3.90)	1.25 (−0.11 to 2.61)	16.22 (3.93)	16.75 (4.97)	0.72 (−0.81 to 2.25)	0.53 (−1.56 to 2.63)	0.613
Phonological fluency (words)	11.00 (5.53)	10.68 (4.75)	0.16 (−0.85 to 1.16)	8.36 (3.41)	8.64 (3.98)	−0.16 (−1.30 to 0.97)	0.32 (−1.23 to 1.87)	0.682
RAVLT-IR (words)	7.04 (1.45)	8.83 (1.58)	1.65 (1.20 to 2.10)	6.12 (1.64)	6.51 (2.04)	0.45 (−0.06 to 0.95)	1.20 (0.51 to 1.89)	0.001
RAVLT-DR (words)	7.08 (2.59)	9.34 (3.17)	2.08 (1.28 to 2.89)	5.58 (2.87)	5.72 (3.42)	0.38 (−0.53 to 1.29)	1.70 (0.46 to 2.94)	0.008

RAVLT-IR: Rey Auditory Verbal Learning Test—immediate recall; RAVLT-DR: Rey Auditory Verbal Learning Test–delayed recall. Values are means ± SDs and differences are means (95% CI). Intragroup comparison by the paired Student’s *t*-test. Intergroup comparison by the Student’s *t*-test. These values are adjusted for retired time and center. Results are based on ANCOVA.

**Table 6:** Changes in quality of life assessed through the EuroQol−5D−3L.

	Intervention Group (*n* = 40)		Control Group (*n* = 40)	
	Baseline	6 Months	Baseline	6 Months
**Mobility**				
No problems in walking about (*n*, %)	34 (85%)	38 (100%)	33 (82.5%)	35 (97.2%)
Some problems in walking about (*n*, %)	6 (15%)	-	7 (17.5%)	1 (2.8%)
Confined to bed (*n*, %)	-	-	-	-
**Self-care**				
No problems with self-care (*n*, %)	40 (100%)	38 (100%)	37 (92.5)	36 (100%)
Some problems washing or dressing (*n*, %)	-	-	3 (7.5%)	-
Unable to wash or dress oneself (*n*, %)	-	-	-	-
**Usual activities**				
No problems with performing their usual activities (*n*, %)	36 (90%)	38 (100%)	33 (82.5%)	36 (100%)
Some problems with performing their usual activities (*n*, %)	4 (10%)	-	7 (17.5%)	-
Unable to perform their usual activities (*n*, %)	-	-	-	-
**Anxiety and depression**				
Not anxious or depressed (*n*, %)	26 (65%)	26 (68.4%)	33 (82.5%)	31 (86.1%)
Moderately anxious or depressed (*n*, %)	14 (35%)	12 (31.6%)	7 (17.5%)	5 (13.9%)
Extremely anxious or depressed (*n*, %)	-	-	-	-
**Pain and discomfort**				
No pain or discomfort (*n*, %)	20 (50%)	18 (47.4%)	17 (42.5%)	15 (41.7%)
Moderate pain or discomfort (*n*, %)	18 (45%)	18 (47.4%)	22 (55.0%)	20 (55.6%)
Extreme pain or discomfort (*n*, %)	2 (5%)	2 (5.2%)	1 (2.5%)	1 (2.7%)

**Table 7:** Changes in the quality of life and functional activities assessed through the EuroQol Scale, WHOQOL-Age test, and Functional Activities Questionnaire (Pfeffer).

	Intervention Group (*n* = 40)			Control Group (*n* = 40)				
	Baseline	6 Months	Change (6 Months-Baseline)	Baseline	6 Months	Change (6 Months-Baseline)	Difference (IG-CG)	*p* Value
EQ-5D-3L score ^a^	0.834 (0.160)	0.847 (0.144)	0.023 (−0.029 to 0.076)	0.828 (0.139)	0.852 (0.122)	0.032 (−0.027 to 0.091)	−0.008 (−0.089 to 0.073	0.839
EQ-VAS EuroQoL visual analog scale ^b^	76.41 (19.03)	75.00 (19.07)	1.27 (−4.15 to 6.70)	74.03 (13.67)	74.17 (15.00)	0.51 (−5.22 to 6.24)	0.76 (−7.35 to 8.88)	0.851
Total score WHOQOL-Age test	50.50 (6.79)	52.29 (5.70)	1.21 (−0.13 to 2.55)	50.57 (5.35)	48.54 (5.58)	−1.39 (−2.93 to 0.15)	2.61 (0.52 to 4.70)	0.015
Total score Functional Activities Questionnaire (Pfeffer)	0.34 (0.82)	0.08 (0.27)	−0.24 (−0.57 to 0.09)	0.19 (0.75)	0.75 (1.65)	0.69 (0.32 to 1.05)	−0.93 (−1.44 to −0.43)	0.001

^a^ Range between 0 (worst quality of life) and 1 (best quality of life). ^b^ Range between 0 (worst quality of life) and 100 (best quality of life). Values are means ± SDs and differences are means (95% CI). Intragroup comparison by the paired Student’s *t*-test. Intergroup comparison by the Student’s *t*-test. These values are adjusted for retired time and center. Results are based on ANCOVA.

## Data Availability

The datasets used and/or analysed during the current study are available from the corresponding author on reasonable request.

## Supplementary Materials

The supporting information can be downloaded at: https://www.mdpi.com/article/10.3390/nu15122811/s1.
