# Supplementary material for: Effects of a Raisin Supplement on Cognitive Performance, Quality of Life, and Functional Activities in Healthy Older Adults—Randomized Clinical Trial
Source: Nutrients. 2023 Jun 20;15(12):2811. doi: 10.3390/nu15122811 (PMC10301980; doi:10.3390/nu15122811)
Supplement: Supplementary file 1 [file nutrients-15-02811-s001.zip › nutrients-2459955-supplementary.pdf]

Supplementary material

General recommendations for a balanced diet

A balanced diet should be varied and contain all kinds of basic foods.

- Consume vegetables, bread and potatoes every day.
- Eat at least three pieces of fresh fruit every day.
- Eat legumes at least three times a week.
- Take olive oil as the main fat in the diet, both for cooking and for dressing.
- Drink milk or low-fat yogurts or cheeses daily.
- Eat fish several times a week, white and blue.
- Fats are necessary for a correct diet but do not exceed the consumption of very fatty animal meats. Choose lean meat without much fat instead and eat it 2 or 3 times a week.
- Do not take high amounts of salt or salty foods. Use garlic, onion, vinegar, lemon, or aromatic herbs for seasoning as an alternative to salt.
- Drink about two liters of water a day.
- You should cut down as much as possible on sweets, pastries, fast food, and soft drinks.
